# Evaluating side effects of nanoparticle‐mediated siRNA delivery to mesenchymal stem cells using next generation sequencing and enrichment analysis

**DOI:** 10.1002/btm2.10035

**Published:** 2016-10-24

**Authors:** Dominic W. Malcolm, Janet E. Sorrells, Daniel Van Twisk, Juilee Thakar, Danielle S. W. Benoit

**Affiliations:** ^1^ Dept. of Biomedical Engineering University of Rochester Rochester NY 14627; ^2^ Center for Musculoskeletal Research, University of Rochester Rochester NY 14642; ^3^ Dept. of Microbiology and Immunology University of Rochester Rochester NY 14627; ^4^ Dept. of Biostatistics and Computational Biology University of Rochester Rochester NY 14642; ^5^ Dept. of Chemical Engineering University of Rochester Rochester NY 14627

**Keywords:** enrichment analysis, mesenchymal stem cells, nanoparticles, off‐target effects, RNAi, RNAseq

## Abstract

RNA interference has immense potential to modulate cell functions. However, effective delivery of small interfering RNA (siRNA) while avoiding deleterious side effects has proven challenging. This study investigates both intended and unintended effects of diblock copolymer nanoparticle (NP) delivery of siRNA delivery to human mesenchymal stem cells (hMSC). Specifically, siRNA delivery was investigated at a range of NP‐siRNA:hMSC ratios with a focus on the effects of NP‐siRNA treatment on hMSC functions. Additionally, next generation RNA sequencing (RNAseq) was used with enrichment analysis to observe side effects in hMSC gene expression. Results show NP‐siRNA delivery is negatively correlated with hMSC density. However, higher NP‐siRNA:hMSC ratios increased cytotoxicity and decreased metabolic activity. hMSC proliferation was largely unaffected by NP‐siRNA treatment, except for a threefold reduction in hMSCs seeded at 4,000 cells/cm^2^. Flow cytometry reveals that apoptosis is a function of NP‐siRNA treatment time and seeding density; ∼14% of the treated hMSCs seeded at 8,000 cells/cm^2^ were annexin V^+^‐siRNA^+^ 24 hr after treatment, while 11% of the treated population was annexin V^+^‐siRNA^−^. RNAseq shows that NP‐siRNA treatment results in transcriptomic changes in hMSCs, while pathway analysis shows upregulation of apoptosis signaling and downregulation of metabolism, cell cycle, and DNA replication pathways, as corroborated by apoptosis, metabolism, and proliferation assays. Additionally, multiple innate immune signaling pathways such as toll‐like receptor, RIG‐I‐like receptor, and nuclear factor‐κB signaling pathways are upregulated. Furthermore, and consistent with traditional siRNA immune activation, cytokine–cytokine receptor signaling was also upregulated. Overall, this study provides insight into NP‐siRNA:hMSC ratios that are favorable for siRNA delivery. Moreover, NP‐siRNA delivery results in side effects across the hMSC transcriptome that suggest activation of the innate immunity that could alter MSC functions associated with their therapeutic potential.

## 1 Introduction

RNA interference (RNAi) is post‐transcriptional gene silencing resulting from homologous base pair interaction of double stranded RNA (dsRNA) molecules and target messenger RNA (mRNA). Multiple types of dsRNA, such as microRNA (miRNA) and small interfering RNA (siRNA), can initiate the RNAi pathway, resulting in sequence‐specific gene silencing through mechanisms that have been reviewed extensively elsewhere.^1^, ^2^, ^3^ The ability to selectively and efficiently silence specific genes based solely on nucleotide sequence makes RNAi‐based drugs promising candidates in myriad applications. This immense therapeutic potential has culminated in over 30 siRNA/miRNA therapeutic clinical trials as of 2015,^4^ with recent studies showing gene knockdown efficacy in humans.^5^

RNAi has recently been recognized as a powerful tool to control tissue‐specific cell differentiation. In particular, use of RNAi to affect mesenchymal stem cell (MSC) differentiation has tremendous potential for tissue engineering applications. Over the last decade, many miRNAs have been shown to regulate MSC differentiation.^6^, ^7^, ^8^ However, translation of RNAi to MSC‐based therapeutic strategies has only recently gained traction due to development of safe and versatile delivery systems that match the needs of clinical translation.^9^ A comprehensive review shows most studies utilized viral transduction or commercial transfection reagents such as Lipofectamine2000.^10^, ^11^ While these studies have provided promising results, the use of viral vectors are controversial due to the risk of mutagenesis^11^ and immunological responses,^12^ and the translational potential of commercial transfection reagents is limited due to proprietary chemistries. Due to these problems, several polymeric siRNA delivery systems have been developed.^13^, ^14^, ^15^, ^16^ For example, we pioneered the development of a self‐assembled diblock copolymer that exhibits excellent gene knockdown in a variety of cells^15^, ^16^, ^17^ with synthetic versatility to introduce targeting moieties^18^ or, in others work, poly(ethylene glycol) to enhance systemic circulation.^19^ Recently, this NP system was shown to modulate gene expression in human mesenchymal stem cells (hMSCs) without causing acute cytotoxicity or affecting MSC differentiation capacity.^16^

Despite outstanding progress in the use of RNAi, adverse effects can result at a variety of levels. Gene knockdown can lead to unanticipated changes in downstream signaling cascades, silencing of partially homologous off‐target genes, activation of the innate immune system and delivery system toxicity.^5^, ^20^ A recent study investigated off‐target effects of 13 different commercial non‐targeting negative control siRNAs from Ambion, Dharmacon, and Qiagen.^21^ Overall, the study concluded that these siRNAs significantly regulate gene expression, and that the extent of differential expression varied among sequences and was cell‐type dependent. In the context of RNAi‐mediated cell differentiation, understanding off‐target effects is especially critical. For example, treatment of MSCs with a commercially available, non‐targeting siRNA resulted in adipocyte differentiation in the absence of typical differentiation factors.^22^ In addition to RNAi off‐target effects, carrier‐mediated toxicities have also been well documented and can be manifested through multiple mechanisms including membrane disruption, generation of reactive oxygen species (ROS), or lysosomal overload in the case of non‐degradable polymers.^23^, ^24^, ^25^ These types of side effects can significantly bias conclusions made regarding therapeutic benefit of siRNA delivery.

While our group has shown effective siRNA delivery to hMSCs with no cytotoxicity or alteration in differentiation capacity using polymeric NPs,^16^ this approach may alter other critical features of cellular function. Therefore, to more deeply examine the polymer‐ and siRNA‐mediated side effects of siRNA‐NP treatment of hMSCs, the objective of this study was twofold: (1) identify treatment conditions that provide for maximal siRNA delivery in hMSCs while maintaining critical cellular functions (e.g., proliferation, metabolic activity, survival), and (2) use next generation sequencing (NGS) to investigate potential side effects via transcriptome‐wide changes in gene expression.

## 2 Results

### 2.1 The effect of cell density on NP‐mediated siRNA uptake

**Figure 1:**
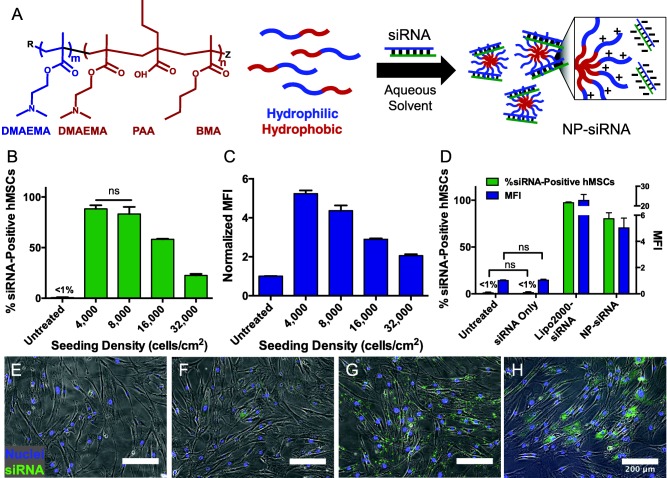
Diblock copolymers self‐assemble into nanoparticles (NP). NP uptake and subsequent gene silencing is a function of hMSC seeding density 24 and 48 hr post‐treatment, respectively. (A) Schematic shows diblock copolymer structure, diblock self‐assembly, and complexation with siRNA. R and Z are functional end groups. *m* = 71, *n* = 138. Abbreviations: DMAEMA = dimethylaminoethyl methacrylate, PAA = propylacrylic acid, BMA = butyl methacrylate. hMSCs were seeded at varying densities and incubated with 30 nM NP‐siRNA complexes. (B) Flow cytometry reveals that a 30 nM dose of fluorescently labeled NP‐siRNA results in significantly greater uptake at lower seeding densities. (C) Relative quantification of the median fluorescent intensity (MFI) of treated cells indicates that the relative amount of NP‐siRNA taken up by cells increases as seeding density decreases. (D) Lipofectamine2000 was used to deliver siRNA as a positive control (Lipo2000‐siRNA) to hMSCs seeded at 8,000 cells/cm^2^. Flow cytometry analysis shows Lipo2000‐siRNA resulted in more siRNA‐positive hMSCs and greater MFI compared to NP‐siRNA. All pairwise comparisons are significant (*p* < .01) unless labeled ns (not significant). Significance was determined using one‐way ANOVA with Tukey's test for multiple comparisons. (E–H) Representative multichannel fluorescence‐phase microscopy corroborates successful NP‐siRNA uptake in hMSCs seeded at 8,000 cells/cm^2^ (H) compared to untreated (E) and siRNA only controls (F). Furthermore, NP‐mediated siRNA delivery results in diffuse siRNA signal throughout the cytoplasm compared to the punctate, confined siRNA signal observed in cells treated with Lipofectamine2000 (G). Scale bar = 200 μm. Error bars represent the standard deviation

Diblock copolymers were synthesized and characterized as previously described^16^ (Supporting Information Table S1) and self‐assembled via dialysis to produce spherical 43 ± 11 nm NP with narrow particle size distribution (PDI = 0.19) and zeta potential of +19 ± 1 mV when measured in 1× phosphate buffered saline (PBS) (Figure 1A, Supporting Information Table S2). Flow cytometry was used to quantify uptake of fluorescently labeled non‐targeting NP‐siRNA complexes delivered to hMSCs at multiple cell seeding densities. Figure 1B shows that the number of MSCs positive for NP‐siRNA uptake significantly increased as MSC seeding density decreased from 32,000 to 8,000 cells/cm^2^. At 32,000 cells/cm^2^, 22% ± 2% of the cell population was positive for NP‐siRNA uptake. This increased to 58% ± 1% at 16,000 cells/cm^2^ and 83% ± 7% at 8,000 cells/cm^2^. The number of siRNA‐positive hMSCs increased to 88% ± 4% when seeding density was decreased to 4,000 cells/cm^2^, but this was not statistically significant compared to 8,000 cells/cm^2^. Furthermore, we analyzed median fluorescence intensity (MFI) of the treated MSCs as a measure of the amount of NP‐siRNA taken up by treated cells. Figure 1C shows MFI increased as seeding density decreased. Although there was no difference in number of siRNA‐positive cells at 4,000 and 8,000 cells/cm^2^, the cells at the lower density exhibited greater overall NP‐siRNA uptake. As a positive control, we treated hMSCs seeded at 8,000 cells/cm^2^ with fluorescent siRNA using Lipofectamine2000 (Lipo2000‐siRNA), a commercially available delivery system. Figure 1D shows that Lipo2000‐siRNA treatment resulted in more siRNA‐positive hMSCs and greater MFI compared to our NP delivery system. To corroborate successful NP‐siRNA uptake, microscopy was used to visualize siRNA uptake (Figure 1E‐H). hMSCs seeded at 8,000 cells/cm^2^ illustrate robust internalization of NP‐siRNA (Figure 1H) that appears diffuse throughout the cytoplasm. Interestingly, Lipofectamine2000 also mediated significant siRNA uptake, however, the internalized siRNA was punctate and confined (Figure 1G), suggesting persistence of siRNA in the endo‐lysosomal compartments.

### 2.2 The effect of hMSC seeding density on NP‐siRNA mediated gene silencing

**Figure 2:**
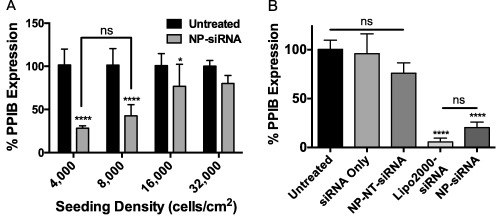
Nanoparticle‐mediated gene silencing was a function of hMSC seeding density 48 hr post‐treatment, and was comparable to Lipofectamine2000. (A) qRT‐PCR shows hMSCs treated with NP‐siRNA targeting peptidylprolyl isomerase B (PPIB) showed significant reduction in *PPIB* gene expression at seeding densities lower than 32,000 cell/cm^2^. (B) NP‐siRNA exhibited gene‐silencing capability that was similar to Lipofectamine2000. *p < .05, ****p < .0001 compared to untreated controls using one‐way ANOVA with Bonferroni's (A) or Tukey's (B) test for multiple comparisons. Error bars represent the standard deviation. ns = not significant. NP‐NT‐siRNA = NPs complexed with a non‐targeting siRNA as a negative control

Gene silencing efficiency of NP‐siRNA treatment was also investigated as a function of seeding density. NPs were complexed with siRNA targeting housekeeping gene peptidylprolyl isomerase B (PPIB) and incubated with hMSCs for 24 hr. *PPIB* mRNA expression was measured 48 hr post‐treatment. Figure 2A shows NP‐siRNA complexes exhibit robust silencing in *PPIB* expression levels in hMSCs seeded at 4,000 and 8,000 cells/cm^2^ achieving gene knockdown to 28% ± 3% and 43% ± 13% of control *PPIB* expression; however, these reductions were not statistically different. Furthermore, hMSCs at 16,000 cells/cm^2^ showed *PPIB* expression of 77% ± 25% relative to untreated controls while gene silencing was attenuated at 32,000 cells/cm^2^.

### 2.3 hMSC function after NP‐siRNA treatments

**Figure 3:**
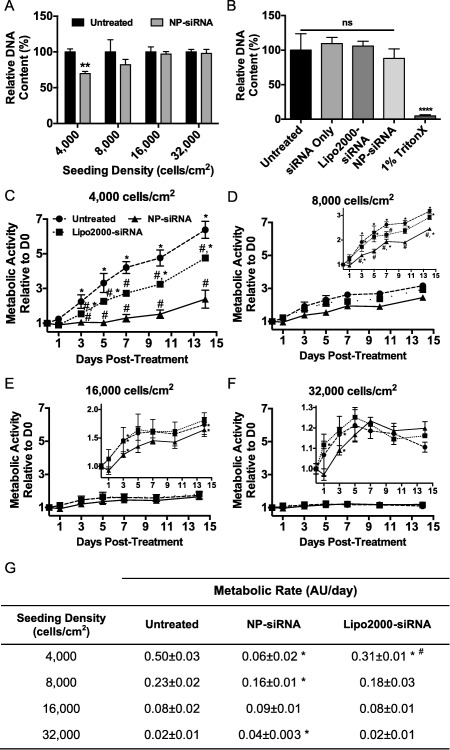
NP‐siRNA treated hMSCs seeded at lower densities showed reduced DNA content 24 hr post‐treatment and reduced cellular metabolism that persisted through 14 days post‐treatment. Quantification of hMSC DNA content suggested significant reduction in hMSC viability when seeded below 8,000 cells/cm^2^ 24 hr post‐treatment (A). At 8,000 cells/cm^2^, the NP system performed similarly to Lipofectamine2000 (B). **p < .01, ****p < .0001 compared to untreated controls determined by one‐way ANOVA with Bonferroni's test for multiple comparisons. Metabolic activity was measured over 14 days in hMSCs that are untreated (•, dashed line), Lipo2000‐siRNA treated (◼, dotted line), or NP‐siRNA treated (▲, solid line) seeded at 4,000 (C), 8,000 (D), 16,000 (E), and 32,000 cells/cm^2^ (F). Insets in (E) and (F) show metabolic activity on a smaller scale to better visualize changes in metabolic activity due to treatment. Linear regression was performed in each metabolic activity curve to quantify metabolic activity to compare among groups (G), * represents significantly different than untreated, and # represents significantly different than NP‐siRNA. (C–F) are representative plots from one experiment that was repeated in an independent experiment that showed similar trends. *p < .05, **p < .01 compared to the previous time point within a single treatment group using two‐way ANOVA with Bonferroni's test for multiple comparisons; ^#^ *p* < .05 untreated compared to treated at a single time point using two‐way ANOVA with Tukey's test for multiple comparisons. Error bars represent standard deviation. AU = arbitrary units

Immediate cytotoxicity and long‐term effects on hMSCs were also examined as a function of NP‐siRNA treatment using non‐targeting negative control siRNA. As a measure of relative hMSC number, DNA content was quantified and reported relative to untreated controls 24 hr post‐treatment. Figure 3A shows NP‐siRNA complexes did not cause significant loss in cell viability at densities from 32,000 to 8,000 cells/cm^2^, as evidenced by unchanged DNA content relative to untreated controls. At 4,000 cells/cm^2^, DNA content in NP‐siRNA treated hMSCs was significantly reduced to 70% ± 3% compared to untreated hMSCs. Figure 3B shows the NP‐siRNA delivery system performed similarly to Lipofectamine2000.

After assessing the immediate effects of NP‐siRNA treatments on hMSC survivability, long‐term metabolic activity of hMSCs was probed. Figure 3C‐F shows metabolic activity for untreated hMSCs and hMSCs treated with NP‐siRNA or Lipo2000‐siRNA at 4,000 (C), 8,000 (D), 16,000 (E), and 32,000 cells/cm^2^ (F). To compare groups, metabolic rates were extrapolated from linear regions of the metabolic activity data (i.e., the first 7 days, Figure 3G). Analysis revealed NP‐siRNA treated hMSCs seeded at 4,000 and 8,000 cells/cm^2^ showed significantly reduced metabolic rates compared to untreated hMSCs. At 16,000 cells/cm^2^, NP‐siRNA treatment did not alter hMSC metabolic rate. Interestingly, at 32,000 cells/cm^2^, NP‐siRNA treated hMSCs showed significant, albeit slight, increases in metabolic rate, which could be attributed to slight variability in initial seeding densities. Figure 3F shows the overlaid curves are nearly indistinguishable. Lipo2000‐siRNA treated hMSCs exhibited reduced metabolic activity only at 4,000 cells/cm^2^ compared to untreated cells, albeit to a lesser extent than NP‐siRNA‐treated hMSCs at the same density.

### 2.4 Effect of NP‐siRNA treatment on hMSC proliferation

**Figure 4:**
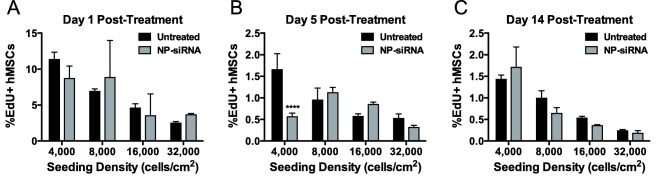
hMSC proliferation was mostly unaltered by NP‐siRNA treatment. There were no significant differences in hMSC proliferation 1 day and 14 days post–treatment (A and C, respectively). However, NP‐siRNA treatment reduced the number of proliferating hMSCs that were seeded at 4,000 cells/cm^2^ 5 days post‐treatment. *n* = 3 from one experiment for (B, C). ****p < .0001 compared to untreated hMSCs using two‐way ANOVA with Bonferroni's test for multiple comparisons. Error bars represent the standard deviation

To better characterize diminished hMSC metabolic activity after NP‐siRNA treatment, hMSC proliferation was measured via 5‐ethynyl‐2′‐deoxyuridine (EdU) incorporation. EdU is a nucleoside analog that is incorporated into newly synthesized DNA during cell proliferation. Figure 4 shows that hMSC proliferation was largely unaltered by NP‐siRNA treatment with a non‐targeting siRNA. However, a threefold reduction in the number of proliferating hMSCs was observed 5 days post‐treatment seeded at 4,000 cells/cm^2^ (Figure 4B). Similar to DNA quantification, these data also show that proliferation is negatively correlated with hMSC seeding density, regardless of NP‐siRNA treatment.

### 2.5 Effect of NP‐siRNA treatment on hMSC apoptosis

**Figure 5:**
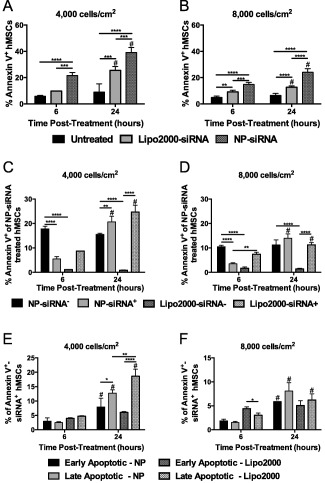
NP‐siRNA treatment initiated modest levels of apoptosis in hMSCs, the extent of which was dependent of hMSC seeding density. NP‐siRNA and Lipo2000 treated hMSCs seeded at lower densities undergo apoptosis to a greater extent than hMSCs at higher densities, and the number of apoptotic cells increases with longer NP‐siRNA or Lipo2000 incubation time (A, B). Of the apoptotic hMSCs treated with NP‐siRNA, the majority was NP‐siRNA^−^ at early time points, while more apoptotic cells were NP‐siRNA^+^ at later timepoints (C, D). Of the apoptotic cells that were NP‐siRNA^+^ after 24 hr NP‐siRNA incubation, there were significantly more late apoptotic than early apoptotic cells. *p < .05, **p < .01, ***p < .001, ****p < .0001 using two‐way ANOVA with Bonferroni's test for multiple comparisons. Error bars represent the standard deviation

Annexin V and propidium iodide (PI) staining of hMSCs was used to measure apoptosis. Specifically, analysis of treated hMSCs seeded at 4,000 and 8,000 cells/cm^2^ was performed via flow cytometry, as hMSC metabolism was reduced at these seeding densities (Figure 5A, B). Generally, annexin V staining increased with time post‐treatment and was greater for NP‐siRNA‐treated cells than Lipo2000‐siRNA treated cells at both seeding densities. At 4,000 cells/cm^2^, 22% ± 2% and 39% ± 4% of NP‐siRNA treated hMSCs were annexin V^+^ at 6 and 24 hr post‐treatment. This is significantly increased compared to Lipo2000‐siRNA treatment and untreated hMSCs. Annexin V staining was ∼50% lower in hMSCs seeded at 8,000 vs. 4,000 cells/cm^2^ in all treatment conditions (Figure 5B). A fluorescently labeled non‐targeting siRNA was used to further discriminate annexin V^+^‐siRNA^+^ hMSCs. At 4,000 cells/cm^2^ 6 hr post‐treatment, only 5% ± 1% of NP‐treated hMSCs were annexin V^+^‐siRNA^+^, while 18% ± 1% were annexin V^+^‐siRNA^−^ (Figure 5C). Nearly 100% Lipo2000‐siRNA treated hMSCs were annexin V^+^‐siRNA^+^. After 24 hr, the number of annexin V^+^‐siRNA^+^ NP‐treated hMSCs increased to 20% ± 2% with 16% ± 0.4% that was annexin V^+^‐NP‐siRNA^−^. The number of Lipo2000‐siRNA treated annexin V^+^‐siRNA^+^ hMSCs significantly increased to 25% ± 3% with no detectable annexin V^+^‐Lipo2000‐siRNA^−^ cells 24 hr post‐treatment (Figure 5C). The same trends were observed at 8,000 cells/cm^2^, but to a lesser extent (Figure 5D). By including PI, a dye impermeable to intact cell membranes, early apoptotic (annexin V^+^‐PI^−^) and late apoptotic (annexin V^+^‐PI^+^) hMSCs could be detected within the annexin V^+^‐siRNA^+^ hMSCs to determine commitment to apoptosis after uptake of siRNA. Figure 5E shows that at 4,000 cells/cm^2^ 6 hr post‐treatment, NP‐siRNA and Lipo2000‐siRNA treatments all resulted in similar amounts of early and late apoptotic‐siRNA^+^ hMSCs. At 24 hr, these populations increased to 8% ± 3% early and 13% ± 1% late apoptotic for NP‐siRNA^+^ hMSCs and 6% ± 0.3% early and 19% ± 2% late apoptotic for Lipo2000‐siRNA^+^ hMSCs. At 8,000 cells/cm^2^ 6 hr post‐treatment results largely corroborated those at 4,000 cells/cm^2^ (Figure 5F) and there were no significant differences between groups at 24 hr.

### 2.6 Transcriptome‐wide effects of NP‐siRNA treatment and gene set enrichment analysis

**Figure 6:**
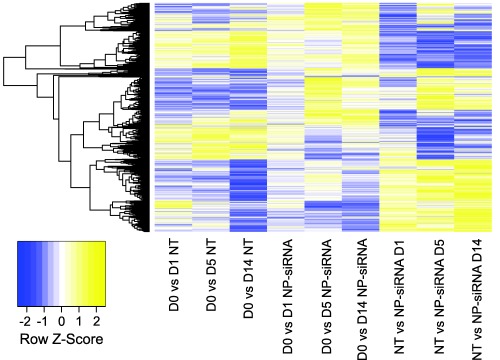
Differentially expressed genes were determined by comparing gene expression levels of conditions described on *x*‐axis (∣log_2_(fold‐change) ∣ > 1 and false discovery rate (FDR) adjusted *p*‐value of .05). Row‐normalized fold‐changes for all differentially expressed genes (rows) are indicated in the heatmap. Abbreviations: D = day; NT = no treatment

Beyond uptake, knockdown, metabolism, proliferation, and apoptosis, RNA sequencing (RNAseq) was performed on hMSCs to observe transcriptome‐wide perturbations in gene expression after delivery non‐targeting siRNA. Reads were mapped to the human reference genome (GRCh38.p2), assembled per gene and condensed into FPKM expression values (fragments per kilobase of transcript per million mapped reads), which provides a measure of expression levels for each gene mapped in the hMSC transcriptome. To visualize transcriptomic changes in untreated hMSCs over time, NP‐siRNA treated hMSCs over time, and NP‐siRNA treated vs. untreated (NT) within each time point, a heatmap was generated showing |log_2_(fold‐change)| > 1 using a false discovery rate adjusted *p*‐value of .05. Figure 6 shows time course analyses in treated and untreated hMSC were nearly identical, except for differences in NP‐siRNA treated hMSCs at day 5 (D5) relative to D0. Additionally, when comparing NP‐siRNA treated vs. untreated hMSCs within each time point, the most drastic changes occur at D5.

**Figure 7:**
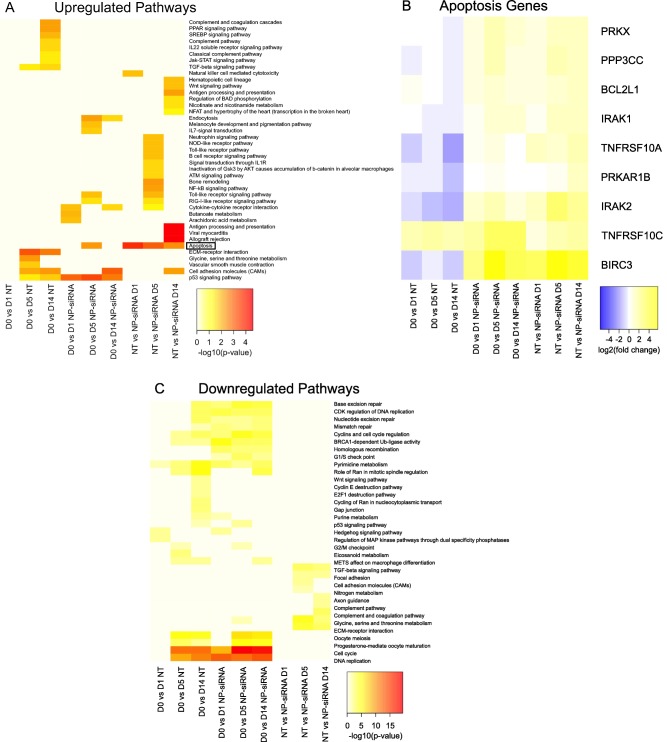
Effect of long‐term culture and NP‐siRNA treatment on the molecular processes and signaling pathways: Up‐regulated (A) and down‐regulated (C) genes in each comparison (*x*‐axis) are enriched in the pathways shown on *y*‐axis. Coloring yellow to red represents low to high enrichment estimated using −log_10_ (*p*‐values). Adjusted *p*‐values are provided in the Supporting Information Table S5. Heatmap of differentially expressed genes within apoptosis (B) enriched signaling pathway, where color refers to log_2_(fold‐change) in expression (blue indicates down regulation and yellow indicates upregulation). Abbreviations: D = day; NT = no treatment

Enrichment analysis was performed on pathways using the DAVID functional annotation tool.^26^ Figure 7A shows upregulated pathways. By comparing day 0 (D0) samples to untreated hMSCs at days 1, 5, and 14 post‐treatment, this analysis reveals pathways that are enriched due to culture conditions alone, such as upregulation of the p53 pathway and ECM‐receptor pathways. To specifically probe the effect of NP‐siRNA treatment, comparisons were made between treated and untreated hMSCs within each time point. As a result of the NP‐siRNA treatment, apoptosis and natural killer cell mediated cytotoxicity pathways are upregulated 1 day post‐treatment. At 5 days post‐treatment, apoptosis signaling is upregulated in addition to multiple pattern recognition receptor signaling pathways, such as Toll‐like (TLR), retinoic acid‐inducible gene 1 (RIG‐I)‐like, and nucleotide‐binding oligomerization domain‐like receptor pathways. Additionally, several inflammatory pathways are upregulated, including cytokine–cytokine receptor interaction and nuclear factor‐κΒ (NF‐κΒ) signaling pathways. Supporting Information Table S6 shows fold‐change expression of multiple signaling molecules from these pathways that are significantly upregulated, such as *TLR3*, *DDX58* (the gene encoding RIG‐I protein), *NFKB1*, *NFKB2*, and various chemokines, cytokines, and associated receptors. By day 14 post‐treatment, apoptosis and immunological pathways such as antigen processing and presentation and allograft rejection pathways are enriched. Figure 7B shows the differentially expressed genes in the upregulated apoptosis pathway, and reveals greater fold‐change upregulation of anti‐apoptotic genes, such as *BIRC3*, *BCL2L1*, and *TNFSF10C*, compared to upregulation of pro‐apoptotic genes, such as *TNFRSF10A*. Figure 7C shows pathways that are significantly downregulated. Cell cycle and DNA replication pathways exhibited the most robust downregulation as a result of culture conditions, not due to NP‐siRNA treatment. Glycine, serine and threonine and nitrogen metabolism pathways were significantly downregulated as a result of NP‐siRNA treatment beginning 5 days post‐treatment. Note that Supporting Information Table S5 contains the adjusted *p*‐values for these enriched pathways.

## 3 Discussion

Outstanding progress has been made in the development of delivery systems for RNAi over the past two decades to unleash its immense therapeutic potential to post‐transcriptionally target specific genes.^13^, ^27^, ^28^, ^29^ We previously developed pH‐responsive diblock copolymers that self‐assemble to form nanoparticles (NP) that are capable of successful siRNA delivery to multiple cell types both *in vitro* and *in vivo*.^15^, ^16^, ^17^, ^30^ Successful gene silencing in MSCs was demonstrated via NP delivery of siRNA without compromising multipotential differentiation capacity.^16^ However, to use this NP delivery system to affect MSC differentiation, it is necessary to thoroughly characterize potential side effects that may abrogate RNAi‐based differentiation signals. Specifically, an extended characterization of how treatment conditions affect NP‐mediated siRNA delivery was performed while comprehensively evaluating potential side effects including MSC metabolism, proliferation, apoptosis, and global RNA expression profiles.

A range of seeding densities was examined to identify how NP‐siRNA:cell ratios affect siRNA delivery, gene knockdown, and identify potential side effects. Results showed that NP‐siRNA delivery was increased at lower seeding densities, likely due to higher NP‐siRNA:hMSC ratios (Figure 1B, C). Higher NP‐siRNA uptake resulted in more robust gene silencing at lower hMSC seeding densities (Figure 2A). Gene silencing capabilities of Lipofectamine2000, a positive control, and NP delivery at 8,000 cells/cm^2^ were statistically equivalent (Figure 2B), even though Lipofectamine2000‐siRNA treatment resulted in greater siRNA uptake (Figure 1D). This suggests that NP delivery of siRNA was more efficient than Lipofectamine2000. This could be explained by the observations made regarding the diffuse nature of the NP‐mediated siRNA delivery in hMSC cytoplasm compared to the punctate signal observed with Lipofectamine2000 (Figures 1H, G), indicating the Lipofectamine2000‐siRNA is confined within endo‐lysosomal compartments.

Although lower seeding densities resulted in greater NP‐siRNA uptake and gene silencing, higher NP‐siRNA:hMSC ratios negatively affected cell viability. Specifically, DNA content was reduced to 70% compared to untreated cells (Figure 3A). This finding might have important implications when adapting this NP‐siRNA delivery system for *in vitro* applications requiring low hMSC densities.^31^, ^32^

When examining unintended side effects of siRNA delivery systems, it is common to measure immediate cytotoxicity. For example, poly(ethylenimine) (PEI) and poly(L‐lysine) are typically used for nucleic acid delivery^33^, ^34^, ^35^, ^36^, ^37^ and have well‐documented cytotoxicity in multiple cell types.^38^ While determining immediate cytotoxicity is necessary, it is equally important to monitor physiological functions in treated cells long term, yet these data are not typically reported. This is critical for applications that require extended culture time beyond initial treatments, including for MSC differentiation. AlamarBlue was used to investigate hMSC metabolic activity over 14 days after NP‐siRNA treatment. AlamarBlue is advantageous over other metabolic assays such as 2,3‐bis‐(2‐methoxy‐4‐nitro‐5‐sulfophenyl)−2*H*‐tetrazolium‐5‐carboxanilide (XTT) and 3‐(4,5‐dimethylthiazol‐2‐yl)−2,5‐(diphenyltetrazolium bromide) (MTT), as it is non‐destructive, allowing for longitudinal measurements across the same cell populations.^39^ Results show that NP‐siRNA treatment reduces metabolic rates of hMSCs at lower seeding densities (Figure 3C, D, G). Lipo2000‐siRNA treatment also resulted in reduced metabolism but to a smaller degree than from NP‐siRNA treatment. These data also show that in untreated samples, metabolic rate decreases as cell seeding density increases. This is likely due to sub‐confluent levels of cell seeding resulting in greater proliferation. For treated samples, it is unknown if reduced metabolic rate is due to alterations in cell proliferation or delayed cell death. AlamarBlue assays have traditionally been utilized to measure cell proliferation^39^; however, metabolic function in bone marrow‐derived stromal cells sometimes does not correlate well with DNA synthesis, which immediately precedes cell division.^40^ To probe cell proliferation directly, an EdU assay was used. Results show that 1 day post‐treatment, NP‐siRNA treatment did not alter hMSC proliferation (Figure 4A); however, NP‐siRNA treatment significantly reduced EdU^+^ hMSCs seeded at 4,000 cells/cm^2^ at 5 days post‐treatment compared to untreated controls (Figure 4B). After 14 days, the hMSCs recovered, and there was no difference in the number of EdU^+^ hMSCs (Figure 4C). These data support AlamarBlue data suggesting that hMSC proliferation is inversely correlated with seeding density, regardless of NP‐siRNA treatment. This could directly explain greater NP‐siRNA uptake at lower seeding densities (Figure 1B, C), as NP uptake is greater in actively proliferating cells.^41^

It is possible that reduction in proliferation 5 days post‐treatment and sustained reduction in metabolic activity could be the result of delayed apoptosis. To investigate this possibility, hMSCs were stained with annexin V and quantified by flow cytometry. Results show that NP‐siRNA treatment resulted in greater numbers of apoptotic hMSCs compared to untreated controls. The extent of apoptotic hMSCs was inversely correlated with seeding density, again most likely due to increased NP‐siRNA to hMSC ratios (Figure 5A, B), possibly through lysosomal overload,^42^ as our polymers are non‐degradable over the time course of this experiment. Lipo2000‐siRNA treatment also resulted in a significant population of apoptotic hMSCs, although to a lesser extent than NP‐siRNA treatment. By performing the analysis with a fluorescently labeled siRNA, approximately half of the apoptotic cells were also positive for NP‐siRNA, which was consistent across all seeding densities (Figure 5D, E), while all of the apoptotic cells treated with Lipo2000 were also siRNA^+^. This suggests that there may be indirect effects of NP‐siRNA treatment that triggers apoptosis. It is possible that the NP‐siRNA^−^ population of apoptotic cells could result from uptake of free/uncomplexed NPs, similar to a PEG‐pDMAEMA‐based siRNA delivery system where free polymer was observed at charge ratios above neutrality, resulting in increased cytotoxicity.^43^ To investigate this possibility, we performed particle‐tracking analysis to quantitatively evaluate siRNA:NP ratio. Results show that NP concentration is 1.13 × 10^12^ ± 2.09 × 10^11^ NPs/mL (Table S2). Using this data combined with siRNA molecular weight, stoichiometry indicates the siRNA:NP ratio is 64 ± 12 for treatments here, suggesting that, statistically, there are no free NPs in treatments that might cause cytotoxicity. Alternatively, apoptotic cells can initiate apoptosis in neighboring cells, as recently described.^44^ Furthermore, the use of PI, a cell impermeable dye, allows for the discrimination of early and late apoptotic cells. Results show that of the apoptotic NP‐siRNA^+^ hMSCs, a significant portion is early apoptotic. This is significant because extracellular exposure of phosphatidylserines, which are detected by annexin V, can be reversible and precedes commitment of apoptosis,^45^ suggesting this population of hMSCs may not ultimately die. Overall, this analysis has shown that only 13% of hMSCs seeded at 4,000 cells/cm^2^ and 8% at 8,000 cells/cm^2^ have committed to apoptosis as a result of NP‐siRNA uptake, while 18% of Lipo‐siRNA^+^ hMSCs at 4,000 cells/cm^2^ were late apoptotic, indicating that Lipo2000‐siRNA treatment results in greater overall apoptosis than the NP.

RNAseq and enrichment analysis were performed to observe transcriptome‐wide perturbations in gene expression and the resulting physiological pathways that might be altered by NP‐siRNA treatment. Results show that apoptosis signaling was the most upregulated pathway due to NP‐siRNA 1 day post‐treatment (Figure 7A). These data are corroborated by results from the annexin V assay that show a significant apoptotic population of hMSCs 1 day post NP‐siRNA treatment (Figure 5B). Interestingly, Figure 7B shows anti‐apoptotic genes, such as *BIRC3*, *BCL2L1*, and *TNFSF10C*, are among those most highly upregulated within the apoptosis pathway, suggesting that cells are recovering from an initial stress due to NP‐siRNA treatment. Additionally, enrichment analysis reveals significant upregulation of multiple innate immune pathways, such as TLR and RIG‐I signaling pathways, both of which are activated by dsRNA molecules.^46^, ^47^ Furthermore this analysis shows upregulation of NF‐κB signaling and cytokine‐cytokine receptor interaction pathways, both of which are downstream of TLR and RIG‐I activation.^46^, ^47^ RNAseq data specifically shows upregulation of *TLR3*, *DDX58* (the gene encoding RIG‐I protein), *NFKB1*, and *NFKB2* 5 days post‐treatment (Table S6). At this same time point, multiple chemokines belonging to the C‐X‐C motif (CXC) and C‐C motif (CC) families and cytokines of the tumor necrosis factor (TNF) and interleukin (IL) families are also significantly altered (Table S6).

Figure 7C shows the most highly downregulated pathways are the cell cycle and DNA replication pathways, but only as a result of culture conditions. This is directly corroborated by EdU results (Figure 4) that indicate hMSC proliferation decreases with time, but was independent of NP‐siRNA treatment at 8,000 cells/cm^2^. Furthermore, multiple metabolic pathways (glycine, serine and threonine and nitrogen metabolism) are downregulated by NP‐siRNA treatment, which is in direct agreement with AlamarBlue results in Figure 3 that show a significant reduction in hMSC metabolism as a result of NP‐siRNA treatment. It is possible that these effects are a result of immune activation; however, there are conflicting reports on whether immune activation decreases^48^ or increases^49^ MSC metabolism.

It has been shown that siRNA can activate the innate immune system via TLR3 interactions.^50^ TLR3 is expressed in the hMSC endosomal compartment^51^, ^52^ making it possible that endosomal trafficking of NP‐siRNA results in TLR signaling. Furthermore, TLR activation in hMSCs has been shown to stimulate secretion of immunomodulatory cytokines and chemokines, such as CCL2, TNF, and multiple interleukins.^51^, ^53^ RIG‐I signaling, which is associated with the immune response, was activated in MSCs after delivery of a double stranded miRNA‐145 mimic using Lipofectamine2000.^54^ This effect was specific to a poly(U) sequence, a dsRNA motif that has been previously shown to activate RIG‐I.^55^ The non‐targeting siRNA pool used in this study contains four non‐targeting siRNAs. Of these, two contain a poly(U) sequence, which could be responsible for upregulation of RIG‐I. RIG‐I signaling could also be activated as a result of TLR crosstalk.^56^ Irrespective of pathway, TLR activation has been shown to affect MSC differentiation in a TLR‐specific manner. Activation of TLR3 via poly(I:C), a dsRNA analogue, inhibited osteogenic differentiation, while activation of TLR4 via lipopolysaccharide enhanced osteogenic differentiation.^57^ Taken together, these results suggest that our NP‐siRNA delivery system may be activating MSC innate immunity at multiple levels: in the endosome where TLR3 receptor is localized when the NPs are endocytosed, and in the cytosol where RIG‐I is located after NP‐mediated endosomal escape of the siRNA. Alterations in this signaling may affect MSC differentiation, although previous work from our lab has shown that siRNA delivery with very similar polymer NPs did not alter MSC adipogenesis, osteogenesis, or chondrogenesis.^16^

To our knowledge, this is the first study probing long term, temporal side effects of nanoparticle‐mediated siRNA delivery in MSCs. Overall, the side effects observed here are consistent with those traditionally associated with siRNA delivery.^5^, ^20^ Furthermore, based on our results, we hypothesize that the use of pH‐responsive polymers for siRNA delivery has the potential to activate the innate immunity at multiple levels via activation of endosomal TLRs and cytosolic RIG‐I‐like receptors. This analysis gives insight into potential side effects of NP‐siRNA delivery, but is limited by convolution of NP and siRNA effects. Separating these effects is especially challenging when using cationic carriers because without shielding by anionic cargo, the delivery system has much different physicochemical properties that can confer unique cellular responses. For example, a previous study has shown that the chain length of poly(dimethylaminoethyl methacrylate) (pDMAEMA), the cationic constituent of the diblock copolymers used here, modulates the mechanism of cytotoxicity from membrane disruption to induction of apoptosis, while the amount of cationic charge influenced the extent and kinetics of endocytosis and intracellular trafficking.^58^ Additionally, in the aforementioned study involving Lipofectamine2000‐mediated delivery of miRNA‐145 in MSCs, treatment with Lipofectamine2000 alone induced an immune response that was unique vs. a negative control siRNA and miRNA‐145.^59^ Unfortunately, use of alternative anionic molecules in place of siRNA has the potential to introduce additional off‐target effects. This, coupled with the fact that naked siRNA cannot be internalized by cells renders NP‐only and siRNA‐only controls inappropriate for this work.

### 3.1 Conclusions

Overall, NP‐siRNA:hMSC ratios have been identified for siRNA delivery while undertaking an extensive characterization of side effects due to NP‐siRNA delivery. An inverse correlation exists between NP‐siRNA uptake and function with hMSC seeding density, while high NP‐siRNA:hMSC ratios can result in cytotoxicity. Furthermore, the importance of monitoring cellular function for extended periods beyond initial treatment is highlighted, as sustained changes in cellular function were observed. Further, NP‐siRNA treatment resulted in a significant portion of annexin V expressing hMSCs that may not be committing to apoptosis. RNAseq and pathway enrichment analysis similarly suggested alterations in hMSC metabolism and proliferation and suggest activation of the innate immune system via TLR and RIG‐I signaling, which could also contribute to apoptosis and changes in metabolism and proliferation. Moreover, this study shows that new technologies, such as NGS coupled with enrichment analysis are powerful tools for identifying potential side effects, which is necessary for safe translation of therapeutic systems from the bench to the clinic.

## 4 Materials and methods

### 4.1 Polymer and nanoparticle synthesis and characterization

Please refer to “Supporting Information.”

### 4.2 hMSC culture

hMSCs were isolated from human bone marrow isolates (Lonza),^60^ and cultured in growth media composed of low glucose Dulbecco's Modified Eagle's Medium (Gibco) with 10% fetal bovine serum (Atlanta Biologics), 1% antibiotic‐antimycotic (Gibco) and 1 ng/mL recombinant human basic fibroblast growth factor‐2 (Corning) and kept at 37°C with 5% CO_2_. hMSCs were used at passage 3–5.

### 4.3 Preparation of NP‐siRNA treatments

NP‐siRNA treatments were prepared at 10× the final treatment concentration of 30 nM siRNA at a charge ratio = 4 (+/−) and added directly to cell culture media, as previously described.^16^ Please refer to “Supporting Information” for description of charge ratio. All untreated samples were given an equal treatment volume of 1× PBS. Where indicated, Lipofectamine 2000 (Invitrogen) was used as a positive control according to the manufacturer's protocol at 30 nM siRNA. All siRNA product numbers and sequences, if available, are listed in Supporting Information Table S3.

### 4.4 Quantification of NP‐uptake via flow cytometry

hMSCs were seeded at 4,000 cells/cm^2^ in 6 well plates and at 8,000, 16,000, and 32,000 cells/cm^2^ in 12‐well plates. Larger plates were used for lower seeding densities to increase overall cell number. NPs were complexed with Silencer FAM‐labeled Negative Control No. 1 siRNA (Ambion) as previously described. After 24 hr incubation, hMSC were washed 3× with 1× PBS, trypsinized and transferred to 1.5 mL tubes and washed once more. hMSCs were resuspended in 100 μL flow buffer (0.5 w/v% bovine serum albumin, and 0.01 v/v% trypan blue to quench extracellular fluorescence^61^) Samples were analyzed on an Accuri C6 flow cytometer. Propidium Iodide (PI, Molecular Probes) was added to each sample (1:500) immediately prior to analysis for discrimination of dead cells. 2,000 hMSC events were gated for analysis. Single stained samples were used for compensation and data was analyzed using FlowJo software.

### 4.5 Microscopy

hMSCs were seeded on Nunc Lab‐Tek II chamber slides (Thermo Scientific) at 8,000 cells/cm^2^ and treated as described previously for flow cytometry. After 24 hr, cells were fixed in 4% paraformaldehyde and mounted with ProLong Gold Antifade Mountant with DAPI (4′,6‐diamidino‐2‐phenylindole, Invitrogen). Phase and fluorescence images were obtained using a Nikon Eclipse Ti2000 inverted microscope.

### 4.6 Assessing NP‐mediated gene knockdown

hMSCs were seeded at 4,000 cells/cm^2^ in 6 well plates and at 8,000, 16,000, and 32,000 cells/cm^2^ in 12‐well plates and treated with NPs complexed with siRNA targeting *PPIB*. After 24 hr, media was replaced and hMSCs were incubated for an additional 24 hr. After 48 hr post‐treatment, RNA was purified using Homogenizer mini‐columns and E.Z.N.A. total RNA Kit I with on‐column DNase digestion (Omega Bio‐tek) according to the manufacturer's protocol. Isolated RNA was quantified and quality was monitored using a NanoVue (GE Healthcare). All 260/280 values were greater than 1.8, indicating high purity RNA. RNA concentration among triplicate samples was normalized, and then reverse transcribed using iScript cDNA Synthesis Kit (Bio‐Rad) according to the manufacturer's protocol. cDNA was diluted to 1 ng/μL RNA equivalence. 2 μL of diluted cDNA was used in each qRT‐PCR reaction. qRT‐PCR was performed using Sso‐Fast EvaGreen Supermix (Bio‐Rad) on a CFX96 Real‐time PCR detection System (Bio‐Rad). Forward and reverse primers were used at 500 nM (sequences are listed in Table S4). qRT‐PCR cycling parameters were as follows: hold at 95°C for 5 min for enzyme activation, then 40 cycles of 95°C for 5 s. denaturing, 55°C (GAPDH) or 60°C (PPIB) for 60 s. annealing, and 72°C for 20 s. extension. Primer efficiencies were calculated from each well as previously described^62^ using 3 and 6% of the maximum amplification to set two thresholds. Relative expression was calculated using the Pfaffl equation relative to untreated samples and normalized to GAPDH expression.^63^

### 4.7 Relative quantification of hMSC DNA content

hMSCs were treated with NPs complexed to ON‐TARGETplus Non‐targeting Control Pool siRNA (Dharmacon) as described previously. 24 hr later, cells were washed twice with 1× PBS and lysed using 1× Luciferase Cell Culture Lysis reagent (Promega) in 1× PBS. Lysates were sonicated for 10 s using a probe sonicator. 10 μL of cell lysates were diluted in 90 μL 1× TE buffer and DNA content was quantified using Quant‐iT PicoGreen dsDNA Assay Kit (Invitrogen) according to the manufacturer's protocol.

### 4.8 Effect of NP‐siRNA treatment on hMSC metabolic activity

Metabolic activity was assessed in treated hMSCs using Alamar Blue Cell Viability Reagent (Molecular Probes) as previously described.^16^ Metabolic activity was normalized to Day 0 samples that were taken immediately before NP‐siRNA treatments were applied.

### 4.9 Quantification of hMSC proliferation after NP‐siRNA treatment

Treated hMSC proliferation was measured using Click‐iT Plus EdU Alexa Fluor 647 Flow Cytometry Assay Kit (Molecular Probes). hMSCs were incubated with 50 μM EdU for 4 hr and staining and detection was carried out according to the manufacturer's protocol. Samples were analyzed on an Accuri C6 flow cytometer. It should be noted that no FAM‐siRNA signal was detectable, in any treated sample, indicating the FAM label may have been compromised during the EdU staining or detection. 5,000–10,000 hMSC events were gated, and FlowJo software was used for analysis.

### 4.10 Quantification of hMSC apoptosis after NP‐siRNA treatment

hMSCs were treated as described in “Quantification of NP‐uptake via flow cytometry.” At 6 and 24 hr post‐treatment, hMSC were washed twice with 1× PBS and the presence of phosphatidylserine residues was detected using an Annexin V Alexa Fluor 647 conjugate (Molecular Probes) according to the manufacturer's protocol. Positive controls for apoptosis were prepared by incubating hMSCs for 20 min at 55°C. PI (Molecular Probes) was added to each sample (1:500 dilution) immediately prior to analysis to enable discrimination of early and late apoptotic cells. Samples were analyzed on an Accuri C6 flow cytometer. 5,000‐10,000 hMSC events were gated, and FlowJo software was used for analysis. Single stained samples were used for compensation and fluorescence minus one controls were used to establish gate boundaries.

### 4.11 Stranded mRNA seq and NGS processing and alignment

hMSCs were seeded at 8,000 cells/cm^2^ in 6 well plates and incubated for 24 hr with NPs complexed with ON‐TARGETplus Non‐targeting Control Pool siRNA (Dharmacon) as described in “Preparation of NP‐siRNA treatments.” RNA was purified as described in “Assessing NP‐mediated gene knockdown” and submitted to the University of Rochester Medical Center's UR Genomics Research Center. RNA concentration was determined with the NanoDrop 1000 spectrophotometer (NanoDrop) and RNA quality was assessed with the Agilent Bioanalyzer (Agilent). Illumina compatible library construction was performed using the TruSeq Stranded mRNA Sample Preparation Kit (Illumina) according to manufacturer's protocols. Briefly, mRNA was purified from 100 ng total RNA with oligo‐dT magnetic beads and chemically fragmented. First‐strand cDNA synthesis was performed using random hexamer priming followed by second‐strand cDNA synthesis using dUTP. End repair and 3′ adenylation was performed on the double stranded cDNA. Illumina adaptors were ligated to both ends of the cDNA, purified by gel electrophoresis and amplified with PCR primers specific to the adaptor sequences to generate amplicons of approximately 200–500 bp in size. The amplified libraries were hybridized to the Illumina single end flow cell and amplified using the cBot (Illumina) at a concentration of 8 pM per lane. Single end reads of 100 nt were generated for each sample and aligned to the human reference genome (GRCh38.p2). Raw reads were generated from an Illumina HiSeq2500 sequencer, and all sequencing data discussed in this publication have been deposited in NCBI's Gene Expression Omnibus (GEO)^64^. All files are accessible through GEO Series accession number GSE87497 (https://www.ncbi.nlm.nih.gov/geo/query/acc.cgi?acc=GSE87497).

### 4.12 Differential expression and pathway analysis

Raw counts were normalized using fragments per kilobase of transcript per million (FPKM). FPKM values less than 10 across all replicated and conditions were removed from further analysis. Differential expression analysis on FPKM normalized counts was performed using Cufflinks version 2.0.2 with the gencode 23 human gene annotations.^65^

Enrichment analysis was performed to identify signaling pathways enriched in the differentially expressed genes DAVID functional annotation tool.^26^ All heat‐maps were plotted using the function “heatmap.2” from the R package gplots.^66^ Adjustment of *p*‐values is performed by Benjamini‐Hochberg wherever indicated. All analysis was performed in R.^67^

### 4.13 Statistical analysis

Each experiment was performed in triplicate in two independent experiments unless otherwise indicated. One‐ or two‐way ANOVA was used with the appropriate post hoc test as indicated to assess significant differences in means (α = 0.05). Statistical analyses were performed using Prism6.0 unless otherwise indicated. For all plots, the mean is represented with standard deviation shown as error bars.

## Conflict of interests

The authors have no conflicts of interest related to this work.

## Supporting information


**FIGURE S1** Critical charge ratio (CR) is determined via gel electrophoresis by loading varying NP:siRNA ratios at different theoretical charge ratios. Critical charge ratio is the CR at which the free siRNA band is absent, indicating complete complexation with NP. (A) Image obtained after running and staining gel under illumination from a UV table. (B) Lane intensity plots produced in ImageJ. Bright bands are represented as negative peaks. (C) Band intensities from Image J and subsequent % siRNA complexation defined as the 100‐(band intensity/free siRNA band inteinsity*100). ND = not detectable
**TABLE S1** Polymer characterization. Verified by GPC^1^, Verified by NMR^2^
**TABLE S2** NP characterization
**TABLE S3** siRNAs used in analysis herein
**TABLE S4** Primer sequences used in this study
**TABLE S5** *p*‐values adjusted using Benjamini‐Hochberg method depicting pathways enriched in upregulated and downregulated genes. See supplementary spreadsheet, “Supplemental_Table_S5.xlsx”
**TABLE S6** Differentially regulated genes involved in innate immune signaling pathways from RNAseq analysis listing official gene symbol, chromosomal locus, log_2_(fold‐change) of NP‐siRNA treated vs. untreated (NT) and FDR adjusted *p*‐value. * = no detectable expression in untreated samples. Italics indicate gene encoding a receptor
