# Supplementary material for: Evaluating side effects of nanoparticle‐mediated siRNA delivery to mesenchymal stem cells using next generation sequencing and enrichment analysis
Source: Bioeng Transl Med. 2016 Oct 24;1(2):193–206. doi: 10.1002/btm2.10035 (PMC5125403; doi:10.1002/btm2.10035)
Supplement: Supplementary file 1 — FIGURE S1 Critical charge ratio (CR) is determined via gel electrophoresis by loading varying NP:siRNA ratios at different theoretical charge ratios. Critical charge ratio is the CR at which the free siRNA band is absent, indicating complete complexation with NP. (A) Image obtained after running and staining gel under illumination from a UV table. (B) Lane intensity plots produced in ImageJ. Bright bands are represented as negative peaks. (C) Band intensities from Image J and subsequent % siRNA complexation defined as the 100‐(band intensity/free siRNA band inteinsity*100). ND = not detectable TABLE S1 Polymer characterization. Verified by GPC1, Verified by NMR2 TABLE S2 NP characterization TABLE S3 siRNAs used in analysis herein TABLE S4 Primer sequences used in this study TABLE S5 p‐values adjusted using Benjamini‐Hochberg method depicting pathways enriched in upregulated and downregulated genes. See supplementary spreadsheet, “Supplemental_Table_S5.xlsx” TABLE S6 Differentially regulated genes involved in innate immune signaling pathways from RNAseq analysis listing official gene symbol, chromosomal locus, log2(fold‐change) of NP‐siRNA treated vs. untreated (NT) and FDR adjusted p‐value. * = no detectable expression in untreated samples. Italics indicate gene encoding a receptor [file BTM2-1-193-s001.docx]

**Supplemental Information**

***Supplementary Methods***

*Synthesis of chain transfer agent (CTA)*

The reversible addition-fragmentation chain transfer (RAFT) polymerization chain transfer agent (CTA), 4-Cyano-4-[(ethylsulfanylthiocarbonyl)sulfanyl]pentanoic acid (ECT), was synthesized as previously described^16,27^.

*Synthesis of cationic siRNA complexation block*

Poly(dimethylaminoethyl methacrylate) was polymerized via RAFT polymerization using ECT and the radical initiator 2,2′-Azobis(2-methylpropionitrile) (AIBN). Distilled DMAEMA was mixed with ECT and AIBN such that the [monomer]/[CTA]/[Initiator] = 100/10/1 in dimethylformamide (DMF) at 25 wt%. The reaction vessel was purged with N_2_ for 40 minutes and polymerized at 60 °C for 6 hours in an oil bath. The reaction was terminated by exposing to atmospheric oxygen. The product was precipitated and washed 4 times in 80:20 petane:diethyl ether with centrifugation and dried under vacuum overnight.

*Synthesis of pH-responsive endosomal escape block*

pDMAEMA macroCTA was added to AIBN and DMAEMA, propylacrylic acid (PAA) and butyl methacrylate (BMA) monomers such that [monomer]/[macroCTA]/[intiatior] = 400/10/1 with relative amounts of DMAEMA:PAA:BMA of 25%:25%:50% which was previously shown to be optimal for endosomal escape ^28^. macroCTA, monomers, and initiator were dissolved in DMF (25 wt% monomers, initiator, and macroCTA to solvent volume). The reaction vessel was purged with N_2_ for 40 minutes and polymerized at 60 °C for 24 hours. The reaction was terminated by exposing to atmospheric oxygen. The product was precipitated in 80:20 pentane:diethyl ether two times and then resolubilized in a minimal volume of acetone. The solubilized product was precipitated and washed three more times in 80:20 pentane:diethyl ether. The polymer was dried overnight under vacuum.

*Polymer characterization*

Absolute molecular weight and polydispersity (PDI, M_w_/M_n_) of pDMAEMA-macroCTA first block and the diblock copolymer were obtained via gel permeation chromatography (GPC) (Shimadzu Technologies) using a TSKgel Guard SuperH-H guard column (Tosoh Biosciences) and a TSKgel Super HM-N for separation using a column oven at 60 °C. The system was equipped with a miniDAWN TREOS multi-angle light scattering detector (Wyatt Technologies), and an Optilab T-rEX differential refractometer (Wyatt Technologies) to determine absolute molecular weights using reported d*n*/dc values for p(DMAEMA) (0.06 mL/g) ^29-31^. HPLC grade DMF containing 0.05 M LiCl was 0.2 μm filtered and used as the mobile phase at a flow rate of 0.35 mL/min. Block copolymer composition was verified using ^1^H-NMR spectroscopy (Bruker Avance400) as previously described^16^. Results of polymer characterization can be found in Table S1.

*NP self-assembly and characterization*

Dried polymer was dissolved in ethanol (EtOH) at a concentration of 4 mg/mL. After complete solubilization, the polymer/EtOH mixture was added to an equal volume of 1x phosphate buffered saline (PBS). This solution was dialyzed in 3,500 Da molecular weight cutoff dialysis tubing against distilled, deionized H_2_O for 24 hours with multiple water changes. The resulting NP solution was then sterile filtered (0.2 μm). To measure concentration, three 1 mL aliquots were distributed in pre-weighed 1.5 mL tubes. The aliquots were frozen, lyophilized, and weighed. The empty tube weight was subtracted from this weight to obtain dried polymer mass from each aliquot to obtain an average polymer concentration. Final NP solution was diluted 10x in 1x PBS for dynamic light scattering analysis (DLS) using a Malvern Zetasizer Nano ZS in disposable cuvettes. Zeta potential was determined using monomodal analysis at the same concentration as DLS measurements in 1x PBS using disposable capillary cells. Nanoparticle concentration was measured using a Malvern Nanosight NS300 Malvern by acquiring 60 s nanoparticle tracking analysis videos in triplicate. Results of NP characterization can be found in Table S2 in the supplemental information.

*Gel retardation assay for critical charge ratio*

The critical charge (CR) ratio is defined as the ratio between the positively charged protonated DMAEMA residues of the pDMAEMA block, where 50% of the residues are protonated at physiological pH, and the negative charges from the siRNA at which there is no free siRNA. This was determined empirically by analyzing several NP:siRNA ratios based on the theoretical charge ratio calculations, and identifying the ratio at which no free siRNA appears via gel electrophoresis (Figure S1). Briefly, 4 μM siRNA (ON-TARGETplus Non-targeting Control Pool, Dharmacon) was incubated with NP to form NP-siRNA complexes at theoretical charge ratios ranging from 0.5-2.0, with no NP used as a control. BlueJuice Gel Loading Buffer (Invitrogen) was used at 1x, and the resulting NP-siRNA samples (20 uL) were loaded on into a 2% agarose gel and run for 45 minutes at 80 V in 1x Tris-Acetate-EDTA (TAE) buffer at pH 7.4. The gel was stained with SybrGold Nucleic Acid Gel Stain (Invitrogen, 1:100 in running buffer) for 30 minutes with gentle rocking. Images were taken of stained gels placed on a UV illumination table using a digital camera (Figure S1A). ImageJ was used to quantify the siRNA band to identify ratios at which complete complexation of siRNA by the NPs occurred (Figure S1B, C). This critical charge ratio was used to determine NP volume necessary to achieve a charge ratio = 4 for NP-siRNA treatments.

|   Charge Ratio: |
| --- |
| **Figure S1:** Critical charge ratio (CR) is determined via gel electrophoresis by loading varying NP:siRNA ratios at different theoretical charge ratios. Critical charge ratio is the CR at which the free siRNA band is absent, indicating complete complexation with NP. (A) Image obtained after running and staining gel under illumination from a UV table. (B) Lane intensity plots produced in ImageJ. Bright bands are represented as negative peaks. (C) Band intensities from Image J and subsequent % siRNA complexation defined as the 100-(band intensity/free siRNA band inteinsity*100). ND = not detectable. |

**Table S1:** Polymer characterization. Verified by GPC^1^, Verified by NMR^2^

| **1st block** | | | **Diblock** | | | **2nd Block Composition^2^** | | |
| --- | --- | --- | --- | --- | --- | --- | --- | --- |
| **Mn^1^ (g/mol)** | **Mw^1^ (g/mol)** | **PDI  (Mw/Mn)** | **Mn^1^ (g/mol)** | **Mw^1^ (g/mol)** | **PDI (Mw/Mn)** | **%DMAEMA** | **%PAA** | **%BMA** |
| 11,100 | 11,400 | 1.03 | 30,400 | 36,200 | 1.19 | 25 | 21 | 54 |

**Table S2:** NP characterization

| **Number Mean  Diameter (nm)** | **PDI** | **Zeta-Potential  (mV)** | **Nanoparticle Concentration  (particles/mL)** |
| --- | --- | --- | --- |
| 43 ± 11 | 0.19 | 19 ± 1 | 1.13x10^12^ ± 2.09x10^11^ |

**Table S3:** siRNAs used in analysis herein

| **Product Name** | **Manufacturer** | **Product #** | **Sequence (5’🡪3’)** |
| --- | --- | --- | --- |
| Silencer FAM-labeled Negative Control No. 1 siRNA | Ambion | AM4620 | Proprietary |
| ON-TARGETplus Cyclophilin B (PPIB) control siRNA (Human) | Dharmacon (GE Healthcare) | D-001820-01-05 | ACAGCAAAUUCCAUCGUGU |
| ON-TARGETplus Non-Targeting Pool | Dharmacon (GE Healthcare) | D-001810-10-20 | UGGUUUACAUGUCGACUAA,  UGGUUUACAUGUUGUGUGA,  UGGUUUACAUGUUUUCUGA,  UGGUUUACAUGUUUUCCUA |

**Table S4:** Primer sequences used in this study

| **Gene** |  | **Nucleotide Sequence (5'**🡪**3')** |
| --- | --- | --- |
| **GAPDH** | Forward | GCAAGAGCACAAGAGGAAGAG |
|  | Reverse | AAGGGGTCTACATGGCAACT |
| **PPIB** | Forward | GTCCGTCTTCTTCCTGCTG |
|  | Reverse | CATCTTCATCTCCAATTCGTAGG |

**Table S5**: p-values adjusted using Benjamini-Hochberg method depicting pathways enriched in upregulated and downregulated genes. See supplementary spreadsheet, “Supplemental_Table_S5.xlsx”

**Table S6:** Differentially regulated genes involved in innate immune signaling pathways from RNAseq analysis listing official gene symbol, chromosomal locus, log2(fold change) of NP-siRNA treated vs untreated (NT) and FDR adjusted p-value. * = no detectable expression in untreated samples. Italics indicate gene encoding a receptor.

| **Signaling Molecules** | |  |  |
| --- | --- | --- | --- |
| **Gene** | **Locus** | **log2(fold change)** | **p_value** |
| DDX58 | chr9:32455704-32526324 | 4.18523 | 0.00E+00 |
| NFKBIE | chr6:44258165-44265788 | 2.83895 | 2.38E-12 |
| TLR3 | chr4:186069151-186088069 | 2.79936 | 4.44E-16 |
| NFKBIA | chr14:35401510-35404749 | 1.96525 | 2.98E-11 |
| IRF7 | chr11:612552-615999 | 1.91945 | 3.88E-10 |
| NFKB1 | chr4:102501328-102617302 | 1.02794 | 1.38E-10 |
| NFKB2 | chr10:102394109-102402529 | 0.987613 | 0.000586799 |
| NFKBIB | chr19:38878554-38908893 | 0.799098 | 0.00290474 |
| **Cytokine, Chemokine, and Receptors** | |  |  |
| **Gene** | **Locus** | **log2(fold change)** | **p_value** |
| CXCL10 | chr4:76011183-76112802 | 1.79769e+308* | 3.80E-37 |
| CXCL11 | chr4:76011183-76112802 | 1.79769e+308* | 1.02E-16 |
| CXCL8 | chr4:73740505-73743716 | 9.02024 | 0 |
| CXCL3 | chr4:74036588-74038807 | 6.49508 | 2.03E-05 |
| *CCR1* | chr3:46163603-46266706 | 5.8339 | -2.86806 |
| CXCL5 | chr4:73995641-73998779 | 4.99875 | 4.02E-08 |
| CXCL12 | chr10:44370164-44386493 | -1.18292 | 7.87E-10 |
| CCL8 | chr17:34319035-34321402 | 1.79769e+308* | 6.80E-05 |
| CCL3 | chr17:36072865-36090169 | 1.79769e+308* | 4.28E-24 |
| CCL5 | chr17:35868966-35885863 | 8.15253 | 2.15E-13 |
| CCL20 | chr2:227813841-227817564 | 4.30096 | 0.00122538 |
| CCL26 | chr7:75769532-75789896 | 1.29296 | 4.17E-08 |
| CCL2 | chr17:34255217-34257203 | 1.04164 | 2.20E-12 |
| IL36B | chr2:113022090-113052867 | 1.79769e+308* | 0.00105629 |
| IL1A | chr2:112773914-112784590 | 5.05842 | 0 |
| IL1B | chr2:112829750-112836903 | 4.52969 | 7.40E-12 |
| *IL13RA2* | chrX:115003974-115019977 | 3.50752 | 2.76E-06 |
| IL32 | chr16:3065296-3087100 | 2.13118 | 0.000570653 |
| *IL7R* | chr5:35852694-35879603 | 1.80012 | 1.94E-05 |
| *IL1R1* | chr2:102064543-102182108 | 1.36252 | 4.92E-05 |
| IL12A | chr3:159913399-160225299 | 0.935676 | 0.00414704 |
| *IL27RA* | chr19:14031747-14053216 | -0.800921 | 0.00356851 |
| *IL11RA* | chr9:34638132-34681298 | -0.983385 | 0.00137222 |
| IL16 | chr15:81159574-81324183 | -1.48542 | 0.00516167 |
| IL26 | chr12:67989444-68234686 | -2.86174 | 0.00278468 |
| TNFSF10 | chr3:172505507-172523507 | 3.72604 | 1.03E-13 |
| TNFAIP6 | chr2:151357591-151380048 | 2.43419 | 1.73E-14 |
| *TNFRSF1B* | chr1:12166942-12209228 | 2.26652 | 0 |
| TNFAIP8L3 | chr15:51037487-51338610 | 2.15567 | 0 |
| TNFAIP3 | chr6:137823672-137883312 | 2.11694 | 7.46E-12 |
| *TNFRSF11A* | chr18:62325286-62391292 | 1.94009 | 0.000765451 |
| TNFSF9 | chr19:6530998-6535928 | 1.48276 | 8.60E-08 |
| *TNFRSF10C* | chr8:23084354-23117437 | 1.4297 | 0.000963464 |
| TNFAIP2 | chr14:103123441-103137439 | 1.32056 | 0.000343343 |
| *TNFRSF14* | chr1:2549919-2565382 | 1.16022 | 7.23E-05 |
| *TNFRSF10B* | chr8:22687658-23083619 | 1.00375 | 4.35E-09 |
| *TNFRSF21* | chr6:47231531-47309905 | -0.48214 | 0.00198156 |
| TNFAIP1 | chr17:28335601-28347009 | -0.775243 | 0.00365218 |
| *TNFRSF10D* | chr8:23135587-23164030 | -0.851381 | 1.04E-10 |
| *TNFRSF11B* | chr8:118923556-118952200 | -1.52136 | 0 |
| *IFNGR1* | chr6:137197483-137219449 | 0.668978 | 2.54E-06 |
